# Supplementary material for: Modelling Skylarks (Alauda arvensis) to Predict Impacts of Changes in Land Management and Policy: Development and Testing of an Agent-Based Model
Source: PLoS One. 2013 Jun 6;8(6):e65803. doi: 10.1371/journal.pone.0065803 (PMC3675089; doi:10.1371/journal.pone.0065803)
Supplement: Supporting Information S4 — The skylark ODdox as a zipped archive. (ZIP) [file pone.0065803.s004.zip › Skylark_ODdox/globals.html]

ALMaSS Skylark ODdox: File Members


ALMaSS Skylark ODdox
 2.0


- Main Page
- Related Pages
- Classes
- Files

- File List
- File Members

- All
- Functions
- Variables
- Typedefs
- Enumerations
- Enumerator
- Macros

- \_
- a
- b
- c
- d
- e
- f
- g
- h
- i
- j
- k
- l
- m
- n
- o
- p
- r
- s
- t
- u
- v
- w
- x

Here is a list of all file members with links to the files they belong to:

### - \_ -

- \_\_3264divide
  : PositionMap.cpp
- \_\_3264minus0
  : PositionMap.cpp
- \_\_3264minus1
  : PositionMap.cpp
- \_\_64bit
  : ALMaSS\_Setup.h
- \_\_CJTDebug\_7
  : skylarks\_all.cpp
- \_\_WEED\_CURVE
  : elements.cpp
- \_CRT\_SECURE\_NO\_DEPRECATE
  : weather.cpp
  , croprotation.cpp
  , plants.cpp
  , pesticide.cpp
  , misc.cpp
  , maperrormsg.cpp
  , rastermap.cpp
  , farm.cpp
  , MovementMap.cpp
  , elements.cpp
  , farm.cpp
  , configurator.cpp
  , hedgebanks.cpp
  , ALMaSS\_CmdLine.cpp
- \_CRTDBG\_MAP\_ALLOC
  : landscape.h
  , PopulationManager.cpp
  , farm.cpp


- Generated on Thu Jan 10 2013 13:15:39 for ALMaSS Skylark ODdox by
   1.8.1.1
